# Functional Homology of WIPI4 and Atg18 Enables *WDR45* Variant Interpretation for BPAN Diagnosis

**DOI:** 10.3390/ijms27157054

**Published:** 2026-08-06

**Authors:** Vincent Morin, Christelle M. Durand, Chloé Angelini, Giovanni Stevanin, Patricia Fergelot, Cyril Goizet, Isabelle Coupry, Jean-Paul Lasserre

**Affiliations:** 1 Bordeaux University, CNRS, INCIA, UMR 5287, NRGen Team, 33076 Bordeaux, France; vincent.morin.1@u-bordeaux.fr (V.M.); christelle.durand@u-bordeaux.fr (C.M.D.); chloe.angelini@chu-bordeaux.fr (C.A.); giovanni-battista.stevanin@u-bordeaux.fr (G.S.); cyril.goizet@chu-bordeaux.fr (C.G.); 2 EPHE, CNRS, INCIA, UMR 5287, 75014 Paris, France; 3 Reference Centre for Neurogenetic Diseases, Department of Medical Genetics, Bordeaux University Hospital, 33000 Bordeaux, France; patricia.fergelot-maurin@chu-bordeaux.fr; 4 Molecular Genetics Laboratory, Department of Medical Genetics, Bordeaux University Hospital, 33000 Bordeaux, France

**Keywords:** BPAN, *WDR45*, *Saccharomyces cerevisiae*, Atg18, PROPPIN

## Abstract

β-propeller protein-associated neurodegeneration (BPAN) is the most prevalent subtype of neurodegeneration with brain iron accumulation (NBIA) and is caused by mutations in the *WDR45* gene encoding the autophagy-related protein WIPI4. However, many *WDR45* missense variants remain classified as variants of uncertain significance (VUS), highlighting the need for reliable functional assays to support their clinical interpretation. In this study, we identified the yeast ortholog of *WDR45* and established a *Saccharomyces cerevisiae*-based functional complementation assay to assess the pathogenicity of *WDR45* variants. We first showed that deletion of the β-propellers that bind polyphosphoinositides (PROPPIN)-encoding genes *ATG18* or *HSV2* causes mitochondrial dysfunction and impaired respiratory growth. Human *WDR45*/WIPI4 specifically rescued the respiratory defect of the *atg18Δ* strain, whereas *WDR45B*/WIPI3 complemented the *hsv2Δ* phenotype, establishing yeast Atg18 as the closest functional counterpart of WIPI4, thus clarifying PROPPIN orthology. We then evaluated a panel of *WDR45* variants and found that benign variants restored normal growth, whereas truncating and pathogenic missense variants failed to complement the *atg18Δ* phenotype, validating the assay for functional variant classification. Finally, we analyzed several VUS identified in patients with clinically compatible BPAN and obtained functional evidence supporting their pathogenicity. Overall, our study establishes a simple, robust, and scalable yeast model that enables functional interpretation of *WDR45* variants and improves molecular diagnosis of BPAN.

## 1. Introduction

Neurodegeneration with Brain Iron Accumulation (NBIA) refers to a clinically and genetically heterogeneous group of rare progressive neurodegenerative diseases whose common hallmark is the iron accumulation in the basal ganglia, as revealed by brain magnetic resonance imaging (MRI) showing T2-weighted signal hypointensity in globus pallidus and substantia nigra [1,2]. This group of diseases is inherited in an autosomal dominant, recessive or X-linked mode. It is characterized by extra-pyramidal symptoms such as dystonia and Parkinsonism, spasticity, associated with neuropsychiatric troubles, optic atrophy or retinal degeneration. Age of onset ranges from childhood to adulthood, with considerable variability in disease progression [3].

To date, 11 genes have been involved in NBIAs and four additional ones in NBIA-like disorders. However, the most frequent clinical-genetic subtypes, accounting for more than 75% of patients, relate to the pantothenate kinase-associated neurodegeneration (PKAN), PLA2G6-associated neurodegeneration (PLAN), mitochondrial membrane protein-associated neurodegeneration (MPAN) and β-propeller protein-associated neurodegeneration (BPAN) forms which are associated with mutations in the *PANK2*, *PLA2G6 C19Orf12* and *WDR45* genes respectively [3]. In terms of pathophysiology, data from the literature suggest that, although iron accumulation plays a role in the pathogenesis of NBIA, it is not considered the primary event in most subtypes [4,5]. Instead, emerging evidence suggests that NBIA may result from an initial combined disruption of key cellular processes, including lipid metabolism, mitochondrial function and autophagy that ultimately lead to iron homeostasis dysregulation [6].

β-propeller protein-associated neurodegeneration, BPAN, also referred to as NBIA5 (OMIM # 300894), is the only X-linked NBIA subtype and accounts for 40 to 45% of all NBIA cases [7,8,9]. The average age of onset of the first symptoms is in early childhood, with overall developmental delay, sleep disturbances and inconstant epilepsy. Neurological deterioration occurs in adolescence or young adulthood with the onset of dystonia, Parkinsonism and cognitive decline. Brain MRI is usually normal during childhood, or with non-specific findings such as corpus callosum atrophy and delayed myelination. Bilateral hypointensities in the substantia nigra and the globus pallidus typically arise during adolescence or young adulthood. BPAN has been shown to result from loss-of-function variants in the *WDR45* gene located on the Xp11.23 chromosome and encoding the WD repeat domain phosphoinositide-interacting protein 4 (WIPI4), one of the four members of the PROPPIN (β-propellers that bind polyphosphoinositides) protein family. WIPIs are highly conserved proteins with a characteristic 7-bladed β-propeller structure. They play a prominent role in autophagy, but also in other membrane trafficking processes [10,11]. Despite the well-established role for WIPI4, the molecular mechanisms underlying BPAN remain largely elusive, which contributes to the absence of specific treatment.

Next-generation sequencing techniques, including whole-exome sequencing (WES) and, increasingly, whole-genome sequencing (WGS), have led to the identification of a growing number of *WDR45* gene variants of all types, establishing BPAN as the most prevalent NBIA subtype. However, many of the identified missense variants are classified as variants of uncertain significance (VUS), posing a significant diagnostic challenge. Indeed, even though in silico analyses are considered a valid tool in clinical practice for interpreting genetic variants, their predictive power is limited and the lack of definitive functional evidence often leaves patients in a prolonged state of diagnostic wandering. Determining whether a VUS is disease-causing remains a crucial hurdle in the clinical management of BPAN and genetic counselling.

Several model systems have been used to study BPAN, including murine model [12,13,14,15,16], zebrafish [17], *Dictyostelium* [18], *Drosophila melanogaster* [19], patients-derived fibroblasts [20,21,22], lymphoblastoid cell lines [23] and induced pluripotent stem cells [24,25]. However, the absence of a clear genotype–phenotype correlation in multicellular organisms including mammalian models complicates the interpretation of variant effects. A simple, genetically tractable system to assess the functional impact of *WDR45* variants would provide an invaluable tool for clinicians and researchers, helping to refine diagnoses and reduce uncertainty for patients.

Despite its apparent simplicity, the yeast *Saccharomyces cerevisiae* (*S. cerevisiae*) remains an extremely useful model organism for exploring gene functions, protein–protein interactions, and genotype–phenotype relationships. Many essential cellular processes are conserved between yeast and humans, with approximately 60% of yeast genes having homologues in humans and 87% of yeast protein domains conserved in human proteins, making yeast highly suitable for translational research [26]. In addition, functional complementation assays using yeast have already been successfully used to assess the pathogenicity of human missense variants, providing a rapid and informative platform [27,28].

Autophagy is a fundamental, evolutionarily conserved degradation and recycling pathway which relies on the orchestrated action of autophagy-related (ATG) proteins [29]. During this process, a double-membrane phagophore originates from the endoplasmic reticulum (ER), expands, engulfing cellular debris such as damaged proteins and organelles, and matures into an autophagosome that fuses with lysosomes, whose enzymes will degrade the enclosed cellular cargo. Among the core ATG proteins are members of the PROPPIN family, namely (WIPI)1-4 proteins in mammals and Atg18, Atg21, and Hsv2 proteins in yeast. These proteins share conserved structural features and likely functional roles in the autophagy pathway. Phylogenetic analyses group Atg18, Atg21, and WIPI1–2 in one clade, while Hsv2 is associated with WIPI3–4 in another. However, multiple studies suggest that WIPI4 is functionally more akin to Atg18 than to Hsv2. Indeed, numerous studies have shown that WIPI4, like Atg18, is essential to the autophagy process. In yeast, Atg2 forms a complex with Atg18 that binds PI3P via the conserved FRRG motif. This complex mediates tethering between the endoplasmic reticulum and the phagophore and transfers bulk lipids, allowing the autophagosome to expand [30]. The same type of structure and interaction has been described for the WIPI4-ATG2 complex in mammals. Through its PI3P-binding capacity, WIPI4 promotes ATG2 anchoring to the phagophore, where ATG2 acts as a lipid channel for phospholipid transfer from the ER to the phagophore, hence promoting phagophore elongation [30,31,32,33,34].

The cellular function of Hsv2 is not as well characterized. In yeast, while it does not appear to be involved in macroautophagy, Hsv2 may play a partial role in microphagy [35,36] and in micronucleophagy [37] and has also recently been implicated in the Golgi membrane-associated degradation (GOMED) pathway, an alternative and Atg5-independent autophagy process [38,39]. Interestingly, Hsv2 shares a slightly higher similarity with WIPI3 than with WIPI4. While WIPI3 is involved in the same GOMED pathway, suggesting that Hsv2 is more likely to be the yeast orthologue of WIPI3, Bueno-Arribas et al. [40] have shown that Hsv2 also binds Atg2 via residues conserved in WIPI4, but not in Atg18, complicating the orthology assignment.

To establish a yeast-based model for BPAN, we first sought to clarify the functional homology between WIPI4 and the yeast Atg18 and Hsv2 PROPPINs. Herein, using the *S. cerevisiae* yeast model, we provided evidence that human WIPI4 is functionally homologous to the yeast Atg18, but not the Hsv2 protein. This yeast model was subsequently validated by functional complementation tests using plasmids expressing either wild-type or mutant versions of *WDR45* or *ATG18*. Finally, we demonstrated the utility of this model for assessing the pathogenicity of several *WDR45* VUS, providing a new tool to support variant interpretation and improve diagnostic accuracy in BPAN patients.

## 2. Results

### 2.1. Yeast Strains Atg18Δ and Hsv2Δ Display a Respiratory Phenotype

As a prerequisite to functional complementation experiments, it was essential to identify a phenotype in the *atg18Δ* and *hsv2Δ* yeast strains that could potentially be rescued by the expression of the human *WDR45* gene. Previous studies have reported that *WDR45* pathogenic variants lead to mitochondrial abnormalities, including alteration of the mitochondrial network, reduced membrane potential, impaired ATP production, and defects in respiratory chain activity, in both cellular and animal models (e.g., fibroblasts, transfected cells and mice) [15,20,41]. Based on these findings, we hypothesized that deletion of *ATG18* or *HSV2* gene in yeast might similarly compromise mitochondrial function, potentially resulting in defective respiratory growth. To test this hypothesis, we assessed the respiratory capacity of the wild-type (WT), *atg18Δ*, and *hsv2Δ* strains by spotting serial dilutions onto media containing either fermentable (glucose) or non-fermentable, respiratory (ethanol or glycerol) carbon sources, at both 28 °C and 36 °C.

**Figure 1:**
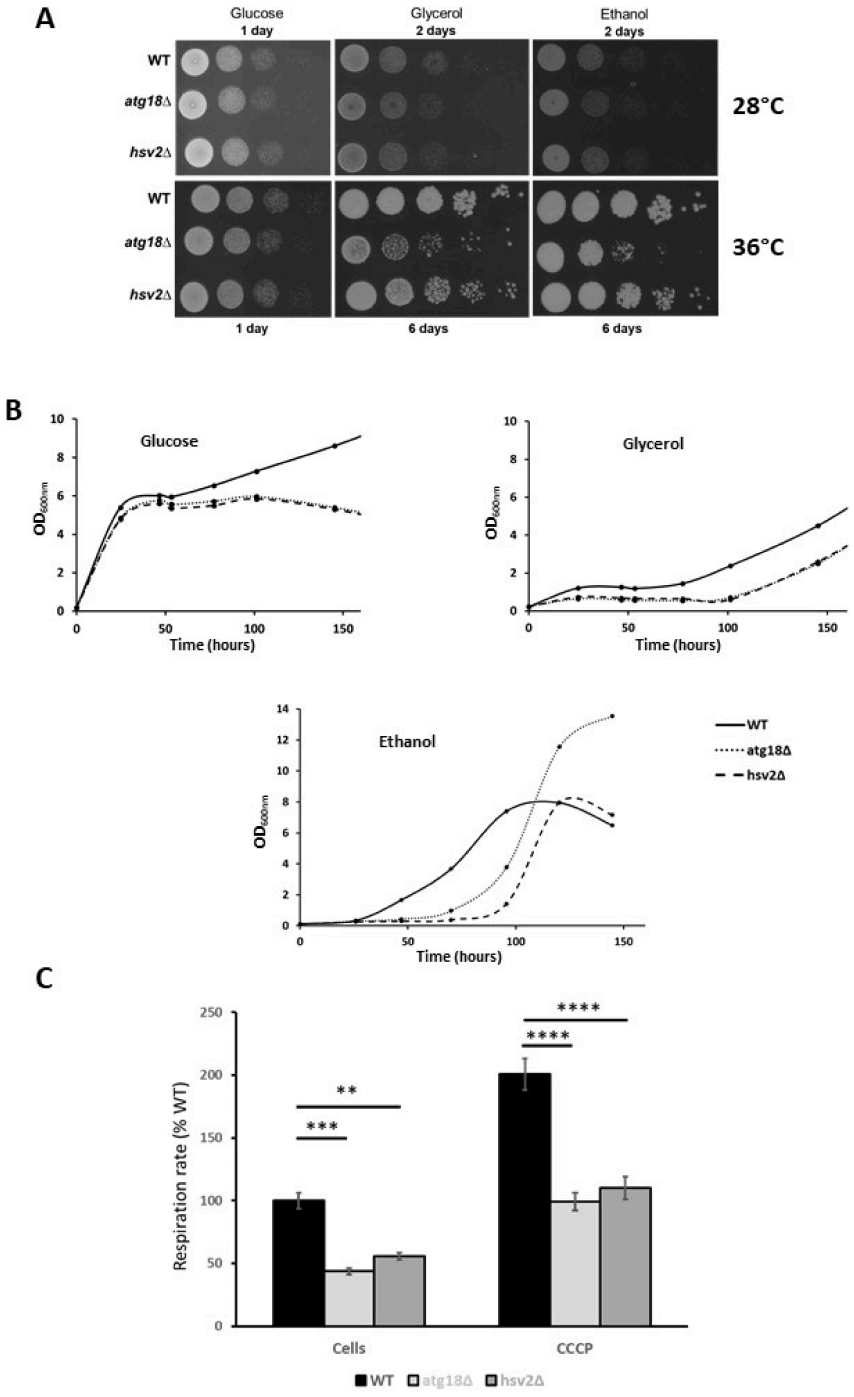
Yeast strains *atg18Δ* and *hsv2Δ* display a respiratory phenotype. (**A**) Cells from wild-type (WT), *atg18Δ* and *hsv2Δ* strains, freshly grown at 28 °C in liquid culture with 2% glucose as carbon source, were serially diluted and spotted onto solid media containing either 2% glucose, ethanol or glycerol. Yeast growth was monitored over time and images were collected after a few days of incubation at 28 °C or 36 °C as indicated. (**B**) Growth rates of WT, *atg18Δ* and *hsv2Δ* strains in liquid media. Yeast cells were inoculated at an optical density of 0.1 OD_600nm_/mL in 25 mL of liquid media containing either 2% glucose, ethanol or glycerol and incubated at 36 °C with shaking over a period of 150h. Optical density measurements at 600 nm were determined once or twice daily. The growth curves are representative of three independent experiments. (**C**) Respiration rate was measured on the same strains grown in liquid medium with 2% glycerol as described in (B). Briefly, when the cells reached an OD_600_ of 1, an aliquot was taken to measure respiration on cells alone or after adding 10 µM CCCP. Respiration values were normalized relative to the wild-type strain. The graphs represent the mean +/− SEM of 3 independent experiments. Statistical analyses were performed using two-way Anova followed by Dunnett’s multiple comparison test. ****: *p* < 0.0001, ***: *p* = 0.0005 and **: *p* < 0.001.

As illustrated in Figure 1A, all strains grew similarly on glucose-containing media, regardless of temperature. However, on respiratory media, both *atg18Δ* and *hsv2Δ* strains exhibited delayed growth compared to the WT, with the impairment being more pronounced at 36 °C. Notably, the respiratory growth defect was more severe in the *atg18Δ* strain than in *hsv2Δ*, suggesting a greater role of Atg18 protein in maintaining mitochondrial function under these conditions.

To confirm and extend these findings, we monitored growth kinetics in liquid cultures using either glucose, glycerol or ethanol as a sole carbon source over a 150 h period (Figure 1B). In glucose-containing media, WT cells exhibited the characteristic diauxic shift—a metabolic transition from fermentative to respiratory metabolism once glucose is depleted. During this shift, yeast cells downregulate fermentation and upregulate mitochondrial pathways to metabolize ethanol produced earlier in fermentation. This transition is typically marked by a transient growth slowdown followed by resumed growth via oxidative phosphorylation. In contrast, *atg18Δ* and *hsv2Δ* strains showed a severely impaired diauxic shift, with markedly reduced growth compared to WT cells once mitochondrial metabolism became essential, further supporting the hypothesis of mitochondrial dysfunction (Figure 1B). Similarly, when cultured directly in a strictly respiratory medium, ethanol or glycerol, as the carbon source, both deleted strains displayed significant growth retardation compared to WT (Figure 1B).

To directly assess mitochondrial function, we measured whole-cell oxygen consumption. In the basal state, both *atg18Δ* and *hsv2Δ* strains retained 44 ± 3% and 56 ± 3% of the respiratory rate relative to WT, respectively. Upon addition of the uncoupler CCCP, which induces maximal respiratory capacity, oxygen consumption increased in both mutant strains, yet remaining approximately 50% lower than in WT cells (Figure 1C). These results are consistent with the observed growth defects on respiratory media.

Together, these findings demonstrate that deletion of *ATG18* or *HSV2* genes in yeast leads to mitochondrial dysfunction, with the *atg18Δ* strain displaying a more pronounced defect. The resulting respiratory phenotypes resemble those seen in human models carrying *WDR45* pathogenic variants, thereby supporting the relevance of these yeast deleted strains as suitable models to investigate functional homology between human WIPI4 protein and its yeast counterparts Atg18 and Hsv2.

### 2.2. Functional Homology Between Human WIPI4 and Yeast Atg18 but Not Hsv2 Proteins

To investigate the potential functional homology between the yeast proteins Atg18 and Hsv2 and the human protein WIPI4, we engineered various yeast strains by introducing plasmids containing either the yeast *ATG18* or *HSV2* genes, or the human *WDR45* gene. Control strains included wild-type, *atg18Δ*, and *hsv2Δ* cells transformed with an empty plasmid (WT + pØ, *atg18Δ* + pØ, and *hsv2Δ* + pØ) to assess any non-specific effects of the plasmid itself on growth. In parallel, the *atg18Δ* and *hsv2Δ* deleted strains were also transformed with plasmids expressing *ATG18*, *HSV2* or *WDR45* genes (*atg18Δ* + p*ATG18*, *atg18Δ* + p*WDR45*; *hsv2Δ* + p*HSV2*, *hsv2Δ* + p*WDR45*).

**Figure 2:**
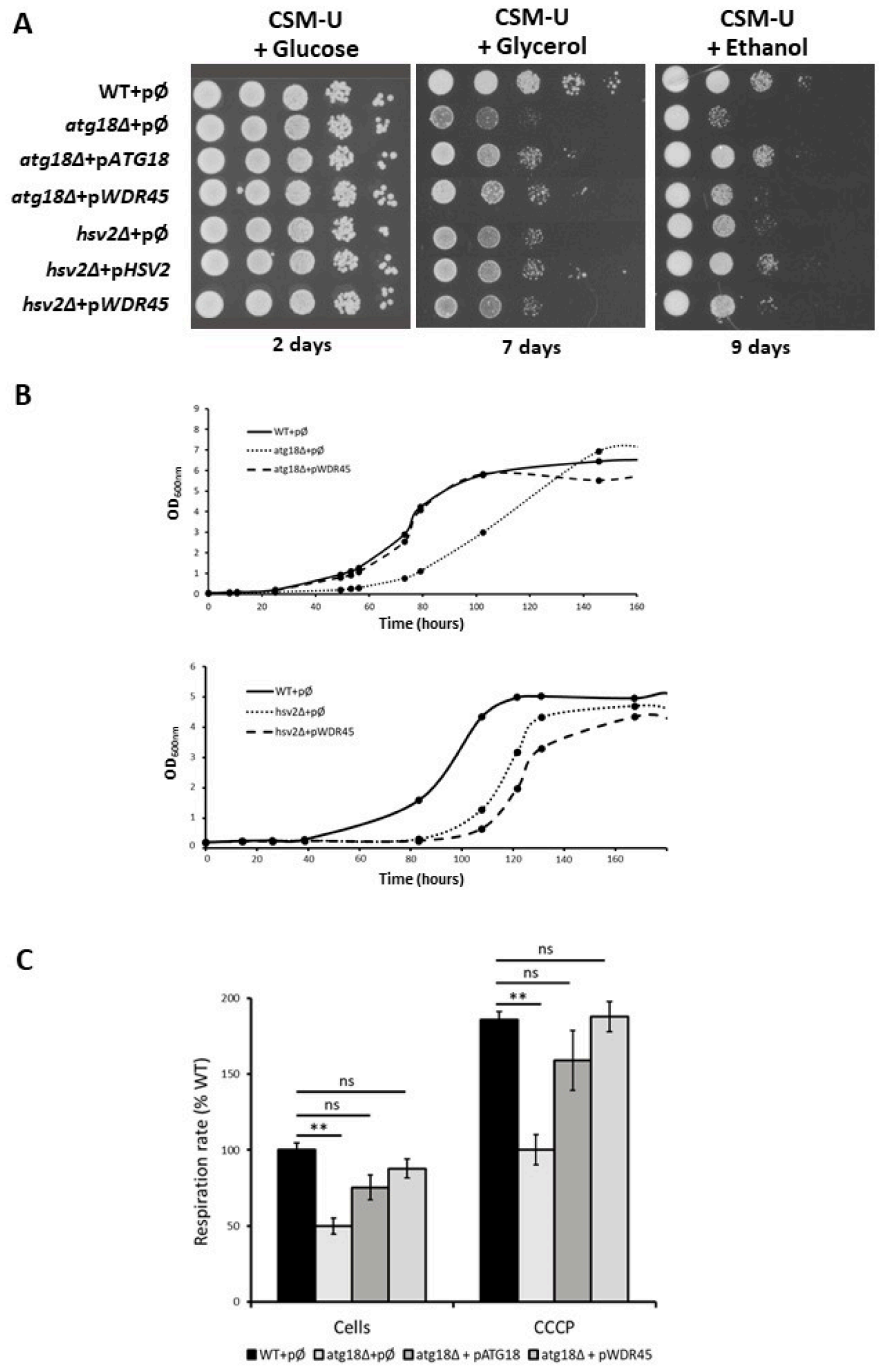
Functional homology between the human *WDR45* gene and yeast *ATG18* or *HSV2* genes. (**A**) Cells from seven strains transformed with either the empty plasmid (WT + pØ, *atg18Δ* + pØ, *hsv2Δ* + pØ) or the plasmid carrying the different genes (*atg18Δ* + p*ATG18*, *atg18Δ* + p*WDR45*, *hsv2Δ* + p*HSV2*, *hsv2Δ* + p*WDR45*) were grown at 28 °C in complete synthetic medium (CSM) with 2% glucose as a carbon source, then serially diluted and spotted onto minimum solid medium without uracil (CSM-U) containing 2% glucose, glycerol, or ethanol. Yeast growth was monitored over time and images were collected after a few days of incubation at 36 °C as indicated. Images are representative of 3 independent experiments. (**B**) Cells from strains transformed with either empty vector (WT + pØ, *atg18Δ* + pØ, *hsv2Δ* + pØ) or vector carrying the two genes (*atg18Δ* + p*WDR45, hsv2Δ* + *pWDR45*), freshly grown in complete synthetic medium without uracil (CSM-U), were inoculated at OD_600nm_ = 0.1/mL in 25 mL of CSM-U with 2% ethanol and incubated at 36 °C with shaking over a period of 160 h. Optical density measurements were determined once or twice daily. The growth curves are representative of three independent experiments. (**C**) Oxygen consumption rate was measured on these cultured strains at an OD_600nm_ = 1. Measures were performed on cells alone or after adding 10 µM CCCP. Values were normalized relative to the WT + pØ strain. The graphs represent the mean +/− SEM of 3 independent experiments. Statistical analyses were performed using two-way Anova followed by Dunnett’s multiple comparison test. **: *p* < 0.001; ns : not significant.

Spot assays on fermentable media revealed that the plasmid had no impact on yeast growth, as no significant differences were observed between WT and WT + pØ (data not shown), or between the deleted strains and their empty vector controls (Figure 2A).

As expected, all strains, regardless of plasmid content, grew normally on complete synthetic media with glucose, at both 28 °C (data not shown) and 36 °C (Figure 2A), indicating the absence of any negative side effects from the plasmid. In contrast, under respiratory conditions (ethanol or glycerol media at 36 °C), the *atg18Δ* and *hsv2Δ* strains harbouring the empty plasmid exhibited reduced growth compared to WT, with the *atg18Δ* strain showing a more pronounced defect, as shown earlier. Importantly, reintroduction of the native *ATG18* or *HSV2* gene into their respective deleted strains restored nearly normal growth confirming that the observed growth defects were specifically due to the absence of these genes. Furthermore, introducing the human *WDR45* gene into the *atg18Δ* background partially restored the respiratory growth phenotype to a level nearly identical to that seen with the native *ATG18* gene, while no rescue was observed in the hsv2Δ background as no improvement in growth across serial dilutions was observed. Taken together, these findings strongly support the idea that *ATG18*, but not *HSV2*, functions as the yeast homolog of human *WDR45* (Figure 2A).

To reinforce these results, we monitored growth of the transformed strains in liquid ethanol-based minimal medium at 36 °C. Consistent with solid media observations, the *atg18Δ* + pØ strain displayed reduced growth relative to WT, a defect that was fully rescued by *WDR45* expression. Conversely, the growth delay in the *hsv2Δ* strain is not rescued by the introduction of the *WDR45* gene (Figure 2B). These data confirm both the respiratory deficiency in the *atg18Δ* mutant and the ability of *WDR45* to complement this phenotype.

Finally, we measured mitochondrial function by assessing whole-cell oxygen consumption. In basal conditions, the *atg18Δ* mutant exhibited approximately 50% lower oxygen consumption than WT. This respiratory defect was largely corrected by expressing either *ATG18* or *WDR45*. Upon CCCP treatment, which uncouples oxidative phosphorylation, respiration increased in all strains; however, the *atg18Δ* mutant still showed a 40–50% deficit, while strains expressing *ATG18* or *WDR45* displayed near-normal levels of respiration (Figure 2C). These results further support a strong functional equivalence between ATG18 and WDR45 in maintaining mitochondrial respiratory function.

**Figure 3:**
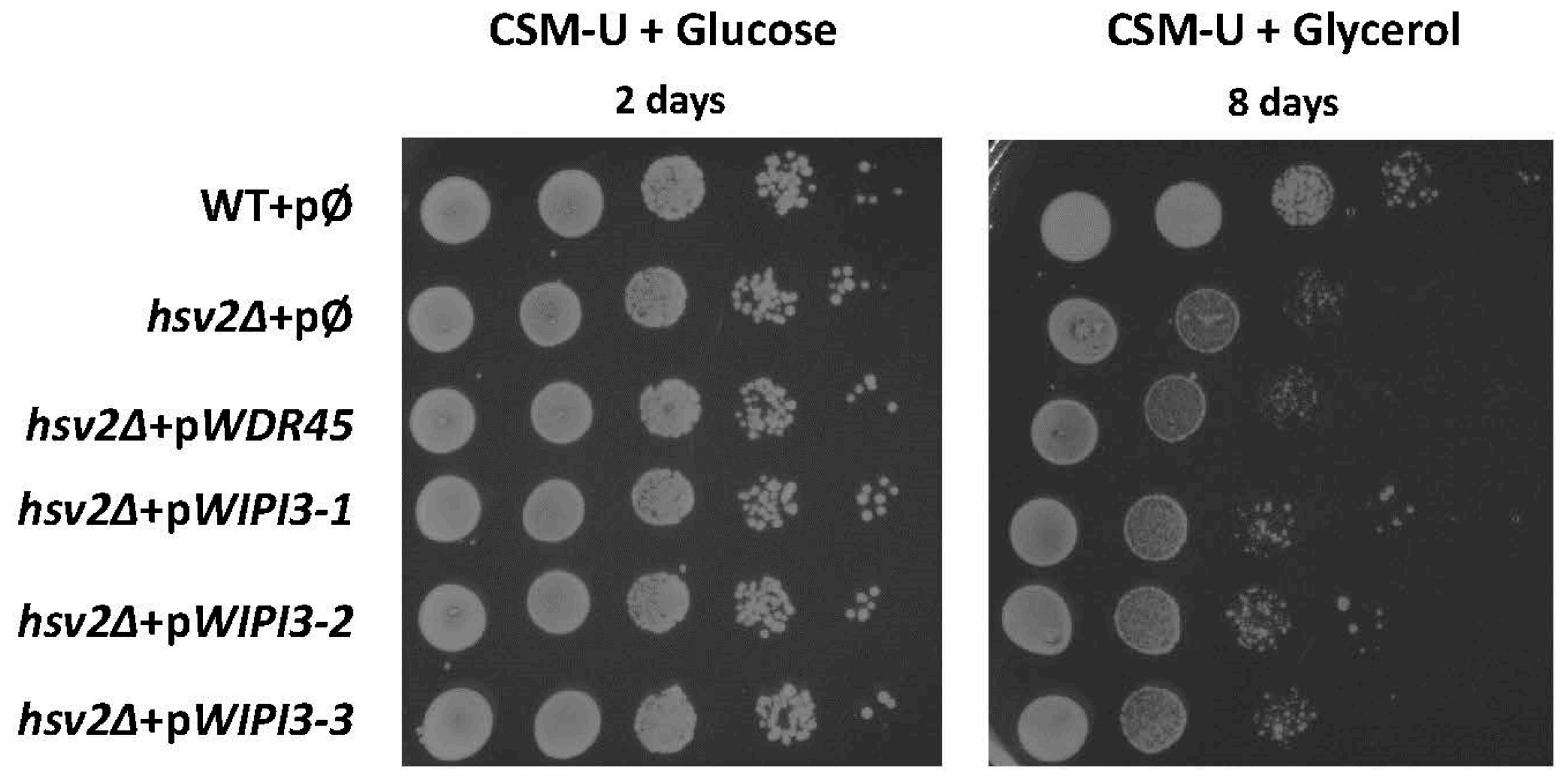
Functional homology between the human *WIPI*3 and yeast *HSV2* genes. Cells from six strains transformed with either the empty vector (WT + pØ, *hsv2Δ* + pØ) or the vector carrying *WDR45* or *WIPI3* genes (*hsv2Δ* + p*WDR45* or 3 different clones of *hsv2Δ* + p*WIPI3*_1 to 3), freshly grown at 28 °C in complete synthetic medium (CSM) containing 2% glucose were serially diluted and spotted onto minimum solid media without uracil containing 2% glucose or glycerol. Yeast growth was monitored over time and images were collected after a few days of incubation at 36 °C as indicated. Images are representative of 3 independent experiments.

Yamaguchi et al. [38] reported that WIPI3, rather than WIPI4, serves as the functional mammalian ortholog of the yeast protein Hsv2. To explore this possibility in our model, we conducted drop test assays to assess the growth of the *hsv2Δ* strain transformed with either an empty plasmid, or plasmids expressing human *WDR45* or three different clones of *WIPI3*. Growth was evaluated on solid media to determine the ability of each construct to rescue the respiratory phenotype. As shown in Figure 3, only the plasmids expressing WIPI3 were able to rescue the growth defect of the *hsv2Δ* strain, while *WDR45* had no effect. These results confirm that WIPI3, and not WIPI4, is the functional ortholog of yeast protein Hsv2.

### 2.3. Validation of the Yeast System as a Useful Diagnostic Tool

Given the demonstrated functional homology between human WDR45 and yeast ATG18 genes, we sought to exploit our yeast complementation system as a model to evaluate the pathogenic potential of WDR45 missense variants identified in BPAN-suspected patients. As a first validation step, we introduced into the atg18Δ mutant strain, pWDR45 plasmids carrying various WDR45 variants previously reported as benign (c.251A>G, p.(D84N); c.838G>A, p.(V280M)) or pathogenic (c.508C>T, p.(Q170*)), and assessed their ability to rescue respiratory growth.

**Figure 4:**
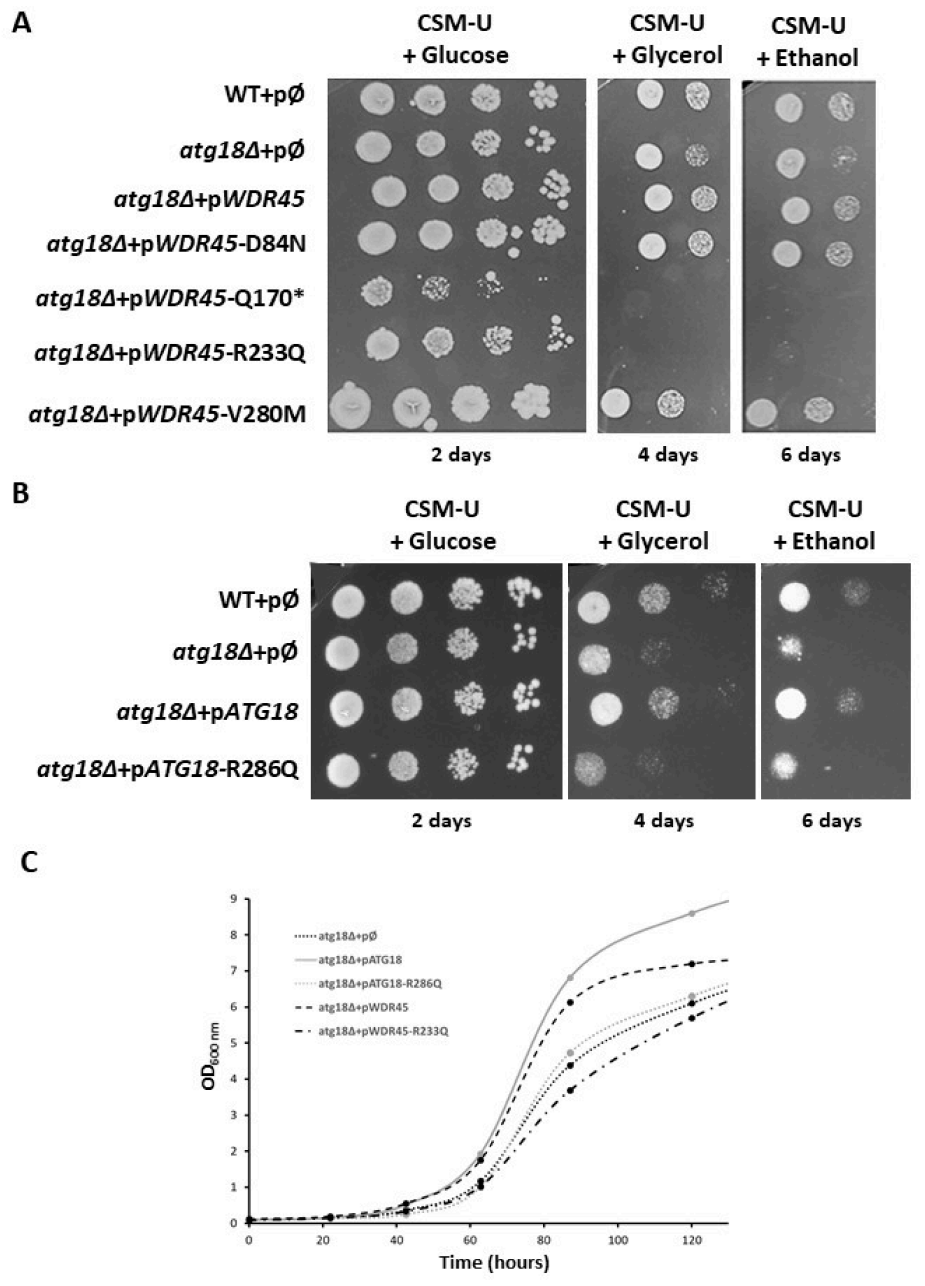
Validation of the yeast system as a useful diagnostic tool. (**A**) Cells from the wild-type and mutant *atg18Δ* strains transformed with either empty vector (WT + pØ, *atg18Δ* + pØ) or vector carrying the *WDR45* gene normal (*atg18Δ* + p*WDR45*) or mutated (*atg18Δ* + p*WDR45*-D84N, p*WDR45*-Q170*, p*WDR45*-R233Q, p*WDR45*-V280M), freshly grown at 28 °C in liquid medium containing 2% glucose were serially diluted and spotted onto solid media containing either 2% glucose, glycerol or ethanol. Yeast growth was monitored over time and images were collected after a few days of incubation at 36 °C, as indicated. (**B**) Complementation of the growth delay phenotype of the *atg18Δ* strain by the *ATG18* gene, normal (*atg18Δ* + p*ATG18)* or carrying a variant affecting a conserved amino acid of the LRRG motif (*atg18Δ* + p*ATG18*-R286Q). The protocol used is identical to that in (**A**). Images are representative of three different experiments. (**C**) Cells from strains transformed with either empty vector (*atg18Δ* + pØ) or vector carrying *WDR45* or *ATG18* genes, normal (*atg18Δ* + p*WDR45*, *atg18Δ* + p*ATG18*) or mutated (*atg18Δ* + p*WDR45*-R233Q, *atg18Δ* + p*ATG18*-R286Q), freshly grown in complete synthetic medium without uracil (CSM-U), were inoculated at OD_600nm_ = 0.1/mL in 25 mL of CSM-U with 2% ethanol and incubated at 36 °C with shaking over a period of 120 h. Optical density measurements were determined once or twice daily. The growth curves are representative of three independent experiments.

As shown in Figure 4A, the nonsense variant Q170* failed to complement the growth defect, clearly indicating a loss of function and supporting its pathogenicity. In contrast, expression of the D84N and V280M variants fully restored respiratory growth of the atg18Δ strain to wild-type level, consistent with their classification as benign. However, these initial missense variants affected residues not conserved in yeast. To further evaluate the predictive power of the yeast model, we also tested a missense variant targeting a highly conserved amino acid residue: c.698G>A (p.R233Q) which affects a conserved arginine within the L/FRRG motif, a domain essential for PI3P binding and autophagosome formation [37,42,43,44]. In line with its predicted deleterious effect, the R233Q variant failed to rescue the respiratory defect of the atg18Δ mutant (Figure 4A), as previously seen with the pathogenic Q170* variation.

To further validate the functional consequences of the R233Q substitution, we introduced the corresponding amino acid change (R286Q) into the yeast ATG18 gene by site-directed mutagenesis. The resulting construct (pATG18-R286Q) was expressed in the atg18Δ mutant background. On fermentable medium, the strain harbouring the mutated gene (atg18Δ + pATG18-R286Q) exhibited growth comparable to both the wild-type strain and the atg18Δ strain complemented with wild-type ATG18. In contrast, when grown on respiratory media (ethanol or glycerol), the mutant strain displayed a marked growth defect, indicating that the R286Q variant was unable to restore mitochondrial function, reproducing the defective phenotype observed with the WDR45-R233Q variant and further supporting its pathogenic nature (Figure 4B).

To strengthen these findings, we evaluated the growth of six yeast strains (WT + pØ, atg18Δ + pØ, atg18Δ + pATG18, atg18Δ + pATG18-R286Q, atg18Δ + pWDR45, and atg18Δ + pWDR45-R233Q) in minimal liquid ethanol medium at 36 °C (CSM-U + ethanol). As expected, the respiratory growth defect of the atg18Δ mutant was improved by the expression of either the wild-type yeast ATG18 or human WDR45 gene. In contrast, neither the mutated yeast gene (pATG18-R286Q) nor the human variant (pWDR45-R233Q) could restore normal growth under these conditions (Figure 4C).

Collectively, these results confirm the pathogenic nature of the R233Q missense variant and further validate the yeast complementation assay as a robust and informative model for the functional characterization of WDR45 variants identified in BPAN patients.

### 2.4. Using the Yeast System to Determine the Pathogenicity of WDR45 Missense Variants Found in BPAN Patients

With a validated and robust yeast complementation model in place, we proceeded to evaluate the functional impact of four WDR45 variants reported in clinical databases (HGMD, ClinVar.), or identified in suspected BPAN patients: three missense variants (c.290T>C, p.(Leu97Pro); c.296T>C, p.(Phe99Ser); and c.500G>T, p.(Gly167Val)) and one in-frame deletion (c.749_751del, p.(Ser250del)). None of the variants were present in GnomAD. The variants L97P, F99S and G167V were classified as uncertain significance by in silico analyses (class 3; PM1, PM2, PP3, according toForgot to define the abbreviation American College of Medical Genetics and Genomics (ACMG) guidelines) [45]. Only the Ser250del variant was listed in ClinVar as pathogenic/likely pathogenic and classified as such according to ACMG guidelines (class 4, PM1, PM2, PM4, PP5). This variant has been reported as de novo in several unrelated individuals [46,47,48]. Amino acids L97 and F99 are located within the third WD repeat of WIPI4, while residues G167 and S250 are found in WD repeats 4 and 6 respectively, and only one of them (G167) is evolutionarily conserved in yeast.

**Figure 5:**
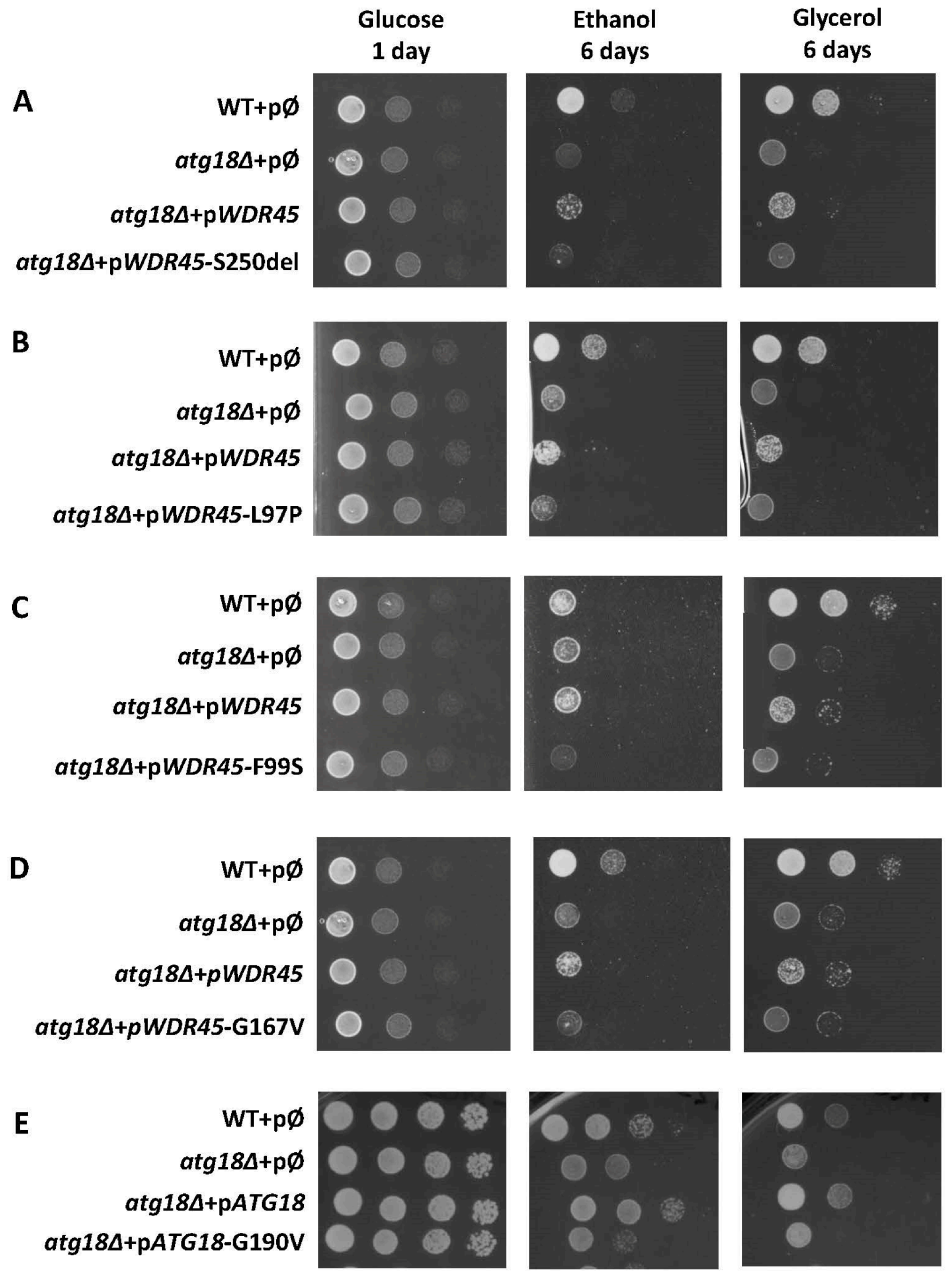
Determination of the pathogenicity of *WDR45* missense variants found in BPAN patients. Cells from the wild-type and mutant *atg18Δ* strains transformed with either empty vector (WT + pØ, *atg18Δ* + pØ) or vector carrying the genes *ATG18* or *WDR45* normal (*atg18Δ* + p*ATG18*, *atg18Δ* + p*WDR45*) or mutated, freshly grown at 28 °C in liquid medium containing 2% glucose were serially diluted and spotted onto solid media containing either 2% glucose or ethanol. Yeast growth was monitored over time and images were collected after a few days of incubation at 36 °C as indicated. (**A**) *atg18Δ* + p*WDR45*-S250del (**B**) *atg18Δ* + p*WDR45*-L97P (**C**) *atg18Δ* + p*WDR45*-F99S (**D**) *atg18Δ* + p*WDR45*-G167V (**E**) *atg18Δ* + p*ATG18*-G190V. Images are representative of three different experiments.

Drop-test assays were performed by expressing each variant in the atg18Δ yeast strain, and the results are presented in Figure 5. Expression of the F99S, L97P, S250del, and G167V variants failed to restore normal growth under respiratory conditions, indicating a loss of function consistent with a pathogenic effect (Figure 5A–D). Notably, since the G167 residue is conserved between WIPI4 and its yeast homolog Atg18, we introduced the corresponding variants (G190V) into the yeast ATG18 gene. As shown in Figure 5E, this mutant allele was unable to complement the respiratory deficiency of the atg18Δ strain, mirroring the loss-of-function phenotype observed with the human G167V variant. These findings provide further functional validation of the G167V pathogenicity.

## 3. Discussion

In this study, we have established a robust yeast-based functional model to investigate BPAN and to support the interpretation of *WDR45* variants, addressing a major unmet need highlighted in the literature. By demonstrating the functional conservation between human WIPI4 and yeast Atg18, we were able to develop tools to clarify the pathogenicity of *WDR45* variants of uncertain significance identified in BPAN patients using mitochondrial defects as a functional complementation test.

One of the central outcomes of this work is the demonstration that human WIPI4/WDR45 is functionally homologous to yeast Atg18, but not to Hsv2. This finding resolves a long-standing ambiguity in the literature regarding PROPPIN orthology. Phylogenetic analyses have consistently separated PROPPINs into two main clades: Atg18/WIPI1–2 and Hsv2/WIPI3–4. Based on sequence similarity alone, WIPI4 clusters more closely with Hsv2 than with Atg18, leading to the hypothesis that Hsv2 might represent the yeast orthologue of WIPI4. However, functional studies have increasingly challenged this assumption [35,49,50,51].

Our complementation assays provide direct functional evidence supporting Atg18 as the true functional counterpart of WIPI4. Expression of human *WDR45* rescued the growth and respiratory defects of the *atg18Δ* strain, but had no effect on respiratory growth phenotype in *hsv2Δ* cell. Conversely, WIPI3—but not WIPI4—rescued the *hsv2Δ* phenotype, in agreement with previous reports linking WIPI3 and Hsv2 to non-canonical autophagy pathways such as GOMED [38]. These results underscore the limitations of phylogenetic inference alone and emphasize the importance of functional assays when assigning orthology, particularly within protein families that have undergone subfunctionalization during evolution.

Taken together, our findings support a model in which Atg18 and WIPI4 share conserved molecular functions related to ATG2 recruitment, PI3P binding, and phagophore expansion, whereas Hsv2 and WIPI3 represent a distinct functional branch of the PROPPIN family.

A second major finding is the identification of a marked respiratory growth defect in *atg18Δ* yeast cells, along with significantly reduced oxygen consumption. This phenotype mimics mitochondrial abnormalities reported in BPAN patient-derived fibroblasts, neuronal models, and animal systems, including disrupted mitochondrial morphology, decreased membrane potential, impaired oxidative phosphorylation, and reduced ATP production [15,20].

Although Atg18 is classically defined as an autophagy protein, our data, along with other studies, suggest that its loss has profound consequences for mitochondrial metabolism [22,52,53]. This is consistent with the intimate links between autophagy, particularly mitophagy, and mitochondrial homeostasis. In yeast, Atg18 is essential for efficient phagophore expansion through its interaction with Atg2, a lipid-transfer protein that supplies membranes required for autophagosome biogenesis. Disruption of this process likely compromises mitochondrial quality control, leading to the accumulation of dysfunctional mitochondria and reduced respiratory capacity.

Importantly, similar mitochondrial phenotypes have been described in BPAN models where autophagic flux is impaired but not completely abolished. This supports the growing view that mitochondrial dysfunction is not merely a secondary consequence of iron accumulation, but a central component of BPAN pathophysiology. Our results therefore align with literature suggesting that BPAN arises from combined defects in autophagy, lipid trafficking, and mitochondrial maintenance, with iron dyshomeostasis emerging as a downstream or amplifying factor rather than the primary cause [17,50].

Another major strength of this study is the validation of a simple, robust, and genetically tractable assay to assess the functional impact of *WDR45* variants. To date, according to the Human Gene Mutation Database (HGMD), 170 *WDR45* variants have been associated with BPAN. While the majority are truncating variants, 20% are missense variants whose pathogenicity remains unclear, presenting a significant challenge for clinical interpretation. These missense variants are frequently classified as variants of uncertain significance (VUS) due to conflicting in silico predictions or inconclusive scoring based on ACMG criteria, and currently, there is no standardized functional assay to assess their impact on WIPI4 activity. Using respiratory growth as readout, we were able to clearly identify the impact of different *WDR45* variants classified as VUS and reclassify them as likely benign or pathogenic based on their functional consequences.

Notably, the model proved informative even for variants affecting residues not conserved between yeast and humans, demonstrating that strict evolutionary conservation is not a prerequisite for functional assessment, thereby validating, within the context of the BPAN, the principle of the well-known humanized yeast model [28].

At the same time, we showed that introducing equivalent variants at conserved residues in yeast *ATG18* reproduced the phenotypes observed with the corresponding human *WDR45* variants. This dual validation, using both the human gene and the yeast orthologue, strongly reinforces the pathogenic interpretation of these variants and highlights the versatility of the system.

Thanks to the widespread use of whole-exome and -genome sequencing, an increasing number of patients are now receiving a molecular diagnosis of BPAN. However, a distinctive feature of this NBIA subtype is its biphasic clinical course. Consequently, the diagnosis cannot be confirmed by MRI during the early stage of the disease, as abnormal iron accumulation is not yet detectable. When sequencing identifies variants of uncertain significance (VUS), patients may remain in diagnostic uncertainty without access to appropriate genetic counselling until they enter the second phase of the disease, which is characterized by the appearance of abnormal iron deposits visible on MRI.

A yeast complementation assay provides functional evidence that can add to in silico predictions and segregation data, thereby helping to refine the classification of variants and contribute to a definitive diagnosis.

Beyond variant interpretation, the yeast model offers a powerful platform for therapeutic discovery. The clear and quantifiable respiratory phenotype associated with *WDR45*/*ATG18* dysfunction is well suited for high-throughput screening of chemical libraries. Compounds that restore respiratory growth or oxygen consumption in the *atg18Δ* strain expressing pathogenic *WDR45* variants could represent candidate molecules capable of bypassing or compensating for autophagy-related defects.

Given the conservation of core autophagy and mitochondrial pathways, hits identified in yeast could serve as a starting point for validation in more complex cellular and animal models. This strategy has proven successful in other neurodegenerative diseases and could accelerate the identification of disease-modifying therapies for BPAN, for which no specific treatment currently exists.

## 4. Materials and Methods

### 4.1. Selection of Variants Studied

In order to validate the yeast model, we first selected variants of the *WDR45* gene whose benign or pathogenic nature had already been established, either because they were present at a high frequency in databases [c.251A>G, p.(Asp84Asn), c.838G>A, p.(Val280Met)], or because they were considered pathogenic as they created a premature stop codon (nonsense variant) [c.508C>T, p.(Gln170*)].

We then selected a variant present in ClinVar and identified in a patient suspected of having BPAN, affecting an amino acid belonging to the L/FRRG motif and highly conserved, including in yeast. This variant, c.698G>A , p.(Arg233Gln), is absent from gnomAD and considered as a likely pathogenic VUS class 4, with the ACMG criteria PM1, PM2, PM5, PP3 [45].

Finally, we selected four variants reported in the HGMD or ClinVar databases and identified in patients presenting with a clinical phenotype and MRI findings suggestive of BPAN. These variants were absent from GnomAD and classified as VUS based on in silico prediction analyses. These included three missense variants [c.290T>C, p.(Leu97Pro); c.296T>C, p.(Phe99Ser); and c.500G>T, p.(Gly167Val)] and one in frame deletion [c.749_751del, p.(Ser250del)]. Among these variants, three had been studied from a functional perspective. Shimizu et al. [50] had analyzed the effect on autophagy of the Phe99Ser, Leu97Pro, and Ser250del variants, but they need to use a HEK293T cell line double knockout for WIPI3 and WIPI4, since single KO cells showed no or only slight phenotype. They were thus able to demonstrate in this model a significant, though not complete, reduction in autophagy for the Leu97Pro variant, near-normal activity for the Phe99Ser variant, and intermediate activity for the Ser250del variant. Furthermore, the impact of the Ser250del variant, reported in three publications [46,47,48], was studied in fibroblasts from a patient carrying the variant. Ferrera et al. [48] showed only a minimal impact on autophagy, but a clear mitochondrial phenotype, notably reduced oxidative phosphorylation and impaired complex I assembly.

To improve readability, unless otherwise stated, the one-letter codes for amino acids will be used throughout the rest of the manuscript.

### 4.2. Yeast Strains, Media and Plasmid Construction

All yeast strains derived from the BY4742 strain, referred to here as WT and presenting the following genotype: MATα his3Δ1 leu2Δ0 lys2Δ0 ura3Δ0. The *hsv2Δ* and *atg18Δ* strains were purchased from the biological resource centers ATCC (Molsheim, France) and Euroscarf (Oberursel, Germany), respectively.

Yeast strains were grown either in rich or minimal media, liquid or solid, depending on the experiment. Rich media contained 1% (*w*/*v*) yeast extract, 1% (*w*/*v*) bactopeptone ((Condalab, Dutscher, Bernolsheim, France), 40 mg/L adenine with 2% carbon source (glucose, ethanol or glycerol) (Sigma Aldrich, St Quentin Fallavier, France).

Defined minimal media consisted of 0.08% (*w*/*v*) Complete Supplement Mixture lacking uracil (CSM–U; Formedium, Norfolk, UK), supplemented with 0.167% (*w*/*v*) yeast nitrogen base without amino acids (Condalab, Dutscher, Bernolsheim, France), 0.5% (*w*/*v*) ammonium sulfate (Acros Organics, Thermofisher Scientific, Illkirch, France), 40 mg/L adenine and 2% carbon source (glucose, ethanol or glycerol).

When required, 2% (*w*/*v*) minimal or rich agar (Sigma Aldrich, St Quentin Fallavier, France), was added for solid media preparation. Media were sterilized by autoclaving at 120 °C for 35 min.

The human and yeast genes were cloned into the pYES2-CT vector (ThermoFisher Scientific, Illkirch, France) containing the *URA3* selectable marker gene.

The human *WDR45* cDNA (NM_001029896.2) synthetized from control human fibroblasts RNA, was amplified by polymerase chain reaction (PCR) using primers containing EcoRI and XhoI restriction sites in their respective 5′ overhangs. The yeast *ATG18* and *HSV2* genes, including approximately 1000 nucleotides of their respective promoter regions, were amplified by PCR from genomic DNA of the wild-type *S. cerevisiae* strain BY4742 with primers containing EcoRI and XhoI restriction sites. Primer sequences are available upon request from the authors.

Purified PCR products were ligated into the EcoRI—XhoI digested pYES2/CT plasmid using T4 DNA ligase (New England Biolabs, Evry, France), and used to transform chemically competent *E. coli* DH5α cells (ThermoFisher Scientific, Illkirch, France). Large-scale plasmid preparation was performed using the NucleoBond^®^ Xtra EF plasmid purification kit (Macherey-Nagel, Hoerdt, France), according to the manufacturer’s instructions. The accuracy of the constructs was verified by Sanger sequencing (Eurofins Genomics, Ebersberg, Germany).

The *WDR45* mutant constructs were generated from the WDR45-pYES2/CT plasmid, using the QuikChange Site-Directed Mutagenesis Kit (Agilent Technologies, Les Ulis, France) and confirmed by direct DNA sequencing (Eurofins Genomics, Ebersberg, Germany).

Yeast strains were transformed by the different plasmid constructs according to the PolyEthylen Glycol PEG-LiAc method and then plated onto appropriate selective media [54]. Transformant colonies were typically visible after 3 days of incubation at 28 °C. We have isolated several independent transformants (3–6 colonies) for each construct to minimize the impact of clone-to-clone variation in plasmid copy number and transgene expression. All transformants carried identical plasmid promoters and selection markers, with the mutation of interest representing the only variable. Because independent transformants are expected to exhibit natural variation in plasmid copy number and expression levels, the consistent observation of the same phenotype across multiple clones provides strong evidence that the phenotype results from the mutation itself rather than from differences in transgene dosage or expression.

### 4.3. Yeast Growth Assay in Solid or Liquid Media

To assess growth phenotypes, serial dilutions were prepared from cultures normalized to an optical density at 600 nm (OD_600_) of 1, yielding dilutions ranging from 10^0^ to 10^−4^. Aliquots from each dilution were spotted onto the appropriate solid media. Plates were incubated at 28 °C and/or 36 °C for several days, depending on the growth rate of the strains and the experimental conditions and pictures were taken every 24h. Drop tests were performed on between 3 and 6 different clones.

Growth kinetics were monitored in liquid medium by measuring optical density at 600 nm at defined time points using a spectrophotometer (Avantor—VWR Int., Rosny, France). Yeast cultures were incubated at 36 °C with continuous shaking at 240 rpm to ensure proper aeration.

### 4.4. Measurement of Respiration Rates in Whole Cells

Yeast cells were harvested during exponential growth phase when the OD_600_ reached 1.0. After centrifugation, cells were resuspended in 1 mL of reaction medium consisting of 1% (*w*/*v*) yeast extract and 1% (*w*/*v*) bactopeptone. Oxygen consumption rates were measured using a Clark-type oxygen electrode (Oxygraph System, Hansatech Instruments, Synersy, Rivesaltes, France) at 28 °C. To assess maximal respiratory capacity, carbonyl cyanide m-chlorophenylhydrazone (CCCP) was added to a final concentration of 10 µM to uncouple oxidative phosphorylation. Oxygen consumption was recorded continuously, and respiration rates were calculated from the linear decrease in oxygen concentration over time. Respiration rates were normalized to cell density and expressed as oxygen consumption per unit of OD_600_.

## Data Availability

The data presented in this study are available upon request from the corresponding author.
